# Investigation of the cerebral blood flow of an Omani man with supposed ‘spirit possession’ associated with an altered mental state : a case report

**DOI:** 10.1186/1752-1947-3-9325

**Published:** 2009-12-10

**Authors:** Amr A Guenedi, Ala'Alddin Al Hussaini, Yousif A Obeid, Samir Hussain, Faisal Al-Azri, Samir Al-Adawi

**Affiliations:** 1 Department of Behavioral Medicine, College of Medicine and Sultan Qaboos University Hospital, Sultan Qaboos University, Muscat, Oman; 2 Department of Radiology and Molecular Imaging, Sultan Qaboos University Hospital, Muscat, Oman

## Abstract

**Introduction:**

The view that spirits may possess humans is found in 90% of the world population, including Arab/Islamic societies. Despite the association between possessive states and various neurological and psychiatric disorders, the available literature has yet to correlate possessive states with functional brain imaging modalities such as single-photon-emission computed tomography.

**Case presentation:**

This paper describes the clinical case of a 22-year-old male Omani patient who presented to us with an altered state of consciousness that his caregiver attributed to possession. We examined whether the patient's mental state correlated with neuro-imaging data. The patient's distress was invariably associated with specific perfusion in the left temporal lobe and structural abnormality in the left basal ganglia.

**Conclusion:**

We discuss the case in the context of possession as a culturally sanctioned idiom of distress, and highlight the importance of studying cross-cultural presentations of altered states of consciousness within biomedical models.

## Introduction

From phrenology to modern neuroscience, there has been a long-standing interest in deciphering the complex relationship between human behavior and brain function. The ultimate aim of such endeavors is to elucidate the underlying biological mechanisms of the development of psychiatric disorders so that evidence-based knowledge on the prevention and management of abnormal behavior can be consolidated [1]. In many areas of clinical medicine, within the central tenet of biomedical models, the linking of signs and symptoms to underlying biological processes is essential Such an achievement has yet to prevail among mental health professionals despite Emil Kraepelin's idealization that psychological disorders are 'housed' within the brain [2]. In the case of altered states of consciousness or abnormal mental states, biomedical models have not yet been proven to be a fruitful approach. It is a commonly held view that psychiatric disorders are amorphous entities and sometimes simply represent an exaggeration of normal psychological processes [3]. It is within these constraints that the modern quest for psychopathology still dwells on descriptive phenomenology, as exemplified by both the Diagnostic and Statistical Manual of Mental Disorders and the International Classification of Diseases [3].

In traditional societies, altered states of consciousness (which would be deemed as manifestations of 'disease' states in psychiatric parlance) are attributed to a state of possession in which a person's behavior is thought to be controlled by an anthropomorphic being that has entered the body [4]. The observed changes in personality tend to vary according to the character of the spirit [4]. A belief that spirits may inhabit human beings is found in 90% of the world's population [5]. Such human-possessing spirits are often blamed for physical and mental disease, and the beliefs and rituals involved in spirit possession constitute culture-specific idioms of distress [5].

To our knowledge, no study has examined whether possessive states can be related to indices of cerebral blood flow. This paper presents a case study of an Omani man who presented with an altered state of consciousness (believed to be caused by spirit possession according to the Omani idiom of distress) and examines whether the patient's dissociative state correlates with functional abnormality in specific regions of the brain. The case is discussed from an anthropological perspective on altered states of consciousness due to supposed spirit possession and the relevance of linking such a phenomenon to a biomedical model.

## Case presentation

A 22-year-old right-handed Omani man first presented to us in 2002. His family brought him into our clinic reporting a history of a recent change in personality and impairment of sensory perception. The patient complained of abnormal auditory experiences when alone. He also complained that the appearance of his father had changed to that of a 'devil'. He claimed that his meals were shared by *Jinn *(evil spirits) which 'made the food taste nasty'. According to the patient's family, the patient had become isolated, disinterested and withdrawn. He had poor sleep with unremitting restlessness.

The patient reported altered attention and concentration coincident with the emergence of his personality change. His personality change had been attributed to various causal agents including supernatural forces such as *Jinn*, contemptuous envy (*Hassad*), the envy-related 'evil eye' ('*Ain*) and sorcery (*Sihr*). He had previously sought traditional treatment for his condition. However, consultation in a traditional healing practice failed to return him to his premorbid self. The family also took him for an *Umra *(optional Muslim pilgrimage to Mecca). Possibly as a result of the stress of traveling, on returning from the *Umra *he became increasingly agitated, which often led to violence towards his family members.

The patient had a positive family history of psychiatric illness: one of his uncles has suffered from symptoms akin to a psychotic illness. In 2001 the patient had been involved in a traffic accident and incurred head trauma, but with no evidence of loss of consciousness or seizures. Immediately after the accident, most of the typical post-concussion syndromes dissipated and he regained physical functionality. About 6 months after the accident, his conduct was noted by the family member to be very different from his premorbid state. He deteriorated in academic competence, which resulted in repeated academic failures and having to leave school. He was noted to be less stressed than normal and his social interaction and self-care regressed drastically to the point at which he was dependent on others for his welfare. This marked deterioration in performing daily living activities coincided with the emergence of auditory hallucinations that came to the attention of the caregivers about 9 months after the accident.

Before seeking consultation with us, he had been seen in two different psychiatric hospitals; he had received electroconvulsive therapy in one of them, but his condition remained impervious to the treatment. During this time, all tests conducted complete blood count, blood biochemistry, immunological workup and electrocardiogram) produced normal results. He sought consultation with an ophthalmologist for double vision, and was diagnosed with retinitis pigmentosa. He was seen by a neurologist for vertigo, double vision, headache and abnormal movements, and was diagnosed with migraine. A computed tomography scan performed at that time showed an encephalomelacia in the left basal ganglia. Electroencephalography suggested possible temporal lobe epilepsy (bilateral with no generalization), but no seizure activity was observed. There were no other abnormal findings. No treatment course had been approved by his family. Because of his obvious personality changes, they continued to attribute his distress to supernatural forces; traditional healing approaches were therefore sought, but they did not improve his condition.

**Figure 1:**
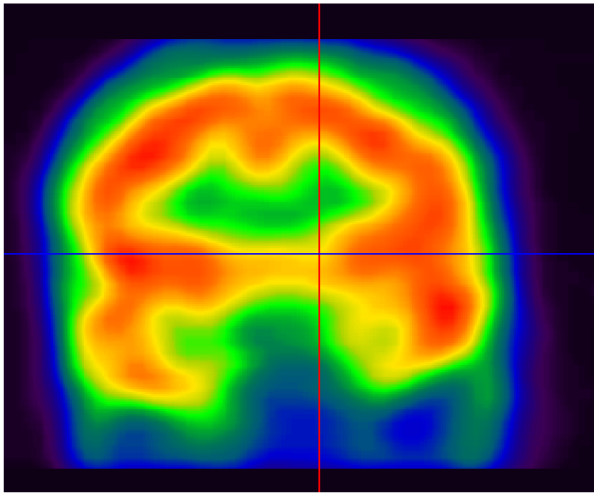
**Pretreatment brain perfusion single-photon-emission computed tomography, showing low perfusion in the left temporal lobe**.

Our preliminary consultation indicated an abnormal temperament, and his social behavior deviated from his culture's social modesty and etiquette. Concurrently, his cognitive functioning was severely compromised. Cognitively, he was inattentive and distractible and showed a strong presence of auditory hallucinations. His psychosocial history did not indicate the presence of alcohol or drug misuse, and physical examination indices were unremarkable. Although there was no indication of receptive and expressive language impairment, he had a disturbance in word generation, suggestive of aphonia and indicative of dysarthria. He was uninterested in maintaining a prosocial behavior and never initiated conversation with others. He remained motionless unless prompted. In formal cognitive testing using the *Folstein Mini-Mental State Exam*, his scores were in the clinically abnormal range, with a total score of less than 19. Blood tests revealed normal complete blood count, blood biochemistry, thyroid functions and lipid levels. Brain perfusion single-photon-emission computed tomography (SPECT) was performed 45 min after injection of a dose of 740 MBq ^99m^Tc-ethyl cysteinate dimer (Bristol-Myers Squibb Medical Imaging) through an existing intravenous line. The image acquisition parameters were 360° of rotation, 64 images, 20s per image with a 128 × 128 pixel matrix [6]. Brain perfusion SPECT was analyzed by an iterative reconstruction method [6]. The indices of tomographic imaging during acute exacerbation of the symptoms are shown in Figure 1; they clearly indicate low perfusion in the left temporal lobe.

**Figure 2:**
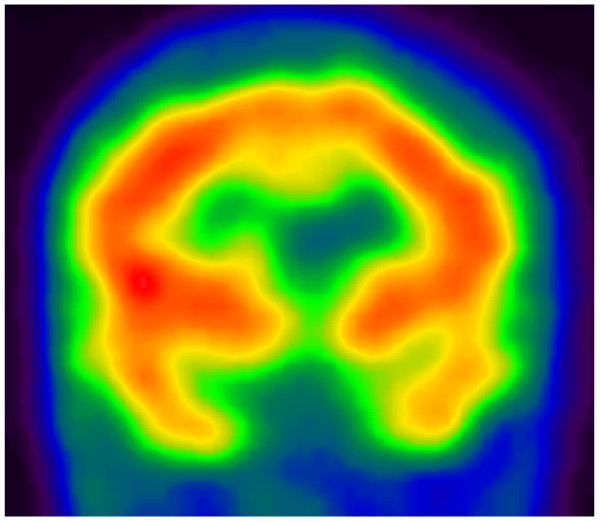
**Post-treatment brain perfusion single-photon-emission computed tomography, showing improvement in perfusion in the left temporal lobe**.

The patient was initially prescribed risperidone (2 mg at bedtime about 3 months later it was combined with lamotrigine (50 mg twice daily). The patient showed a marked improvement in his mood, cognitive functioning, and social behavior after having been on the medications for 3 weeks. His perceptual disorders gradually receded. He relapsed when he was allowed to spend a weekend at home, during which he was not adherent to the medications. After his relapse he was given long-acting intramuscular risperidone in the clinic every 2 weeks. For a period of 4 months. On subsequent follow-ups, he seemed to have returned to his premorbid self. He was well oriented to time and place, was cooperative, and all indicative psychotic features had fully receded. His quality of life had improved, and he had resumed his studies and had progressed in his quest for a certificate-granting secondary school. In addition to these behavioral changes, repeated brain perfusion studies (Figure 2) showed an improvement of perfusion in the left temporal lobe.

## Discussion

The reported case is of a patient who sought psychiatric consultation from tertiary care. After protracted neurological, psychiatric and medical observation, the patient's distress was critically associated with specific functional changes in the temporal lobe and structural abnormality as well as encephalomelacia in the left basal ganglia. After pharmacological intervention, the patient's emotional and cognitive distress eventually receded. The psychological and behavioral improvements coincided with measurable changes in blood perfusion in temporal regions of the brain. Despite the severity of the patient's condition before treatment, his recovery was dramatic but seemingly consistent with available literature. Although the exact mechanisms by which atypical antipsychotic medications (such as risperidone) produce their ameliorative effects remain unclear. Such compounds frequently alleviates symptoms such as those in the presented case (that is, delusions, auditory hallucinations and catatonic behavior) [6-8].

To our knowledge, this is the first case report associating neurobehavioral impairment, neuro-imaging data and a common local idiom of distress in Oman, namely spirit possession. Within traditional Omani society, abrupt personality changes or altered states of consciousness are commonly attributed to spirit possession [4]. The belief in possession is embedded in social- cultural teaching, in which invisible spirits are deemed to inhabit the earth and influence humans by appearing in the form of an anthropomorphic being. In anthropological literature [9,10], possession is classified into three types. The first is the symbiotic type, in which the spirit and the human being have a 'platonic' form of relationship. The second type of possession is a partial possession that is reminiscent of dissociative identity disorders in psychiatric parlance [4,10]. The final type (discussed in this case report) represents total possession, in which a person's behavior is totally controlled by a spirit. Psychiatric interest in possession owes its origin to the writing of Jean-Étienne Esquirol, who described the phenomenology of spirit possessions as 'disease' [11]. Despite similarities between neurologically induced disorders and the 'abnormal behavior' deemed to be triggered by possession, there has yet to be a report linking possession to brain abnormality. This problem is compounded by critiques urging that, even if biomarkers are found for psychological disorders, it will prove to be even more difficult to establish whether such defects are truly representative of the pathology or are simply by-products of a compensatory adaptation to the distressed state [12].

From a biomedical perspective, the condition of the current patient would suggest symptoms of chronic schizophrenia, a diagnosis that is supported by a family history of psychosis. In the parlance of modern psychiatry, the patient met criteria for schizophrenia and responded to risperidone, a known treatment for psychosis. A closer observation of his sustained traumatic brain injury revealed the presence of intransigent and persistent cognitive and behavioral dysfunctions, and poor response to electroconvulsive therapy, which could point to an organic pathology. With the background of observed abnormal electroencephalographic activity in the present case, the possibility remains that lamotrigine may have ameliorated the patient's psychotic symptoms by controlling 'non-convulsive seizures'. It is interesting to note that many patients diagnosed with schizophrenia have a history of traumatic brain injury [13]. From the perspective of the present case, functional (SPECT) and structural neuro-imaging data indicated abnormalities in the left temporal lobe and left basal ganglia, regions that have been shown to accentuate the spectrum of cognitive, emotional and motor disorders, as observed in the present case [14].

By correlating functional brain activation with spirit possession, this case study bridges the gap between cultural phenomena and modern psychiatry. To come to grips with this complex issue, as well as to explain variants of mental illness, Kiev [15] suggested that the 'hardware' or pathology of mental illness can be traced back to brain abnormalities, whereas the phenotypical presentation of the observed 'abnormal behavior' constitutes 'software'. The present study suggests that possessive states - in this context, culture-bound syndromes - may be accompanied by specific neural structural and functional activities that warrant further investigation. SPECT revealed that the patient had a biological illness with two possible diagnoses, schizophrenia or sequelae of traumatic brain injury. There is therefore heuristic value in undertaking more biological research on culture-bound syndromes.

## Conclusions

This case report suggests that culture-bound phenomena, such as spirit possession in Oman, can have a biological basis. Biological studies of patients with culture-bound syndromes should be pursued, to shed light on the possible overlap between culture-bound syndromes and psychiatric disorders described in the Diagnostic and Statistical Manual of Mental Disorders and the International Classification of Diseases.

## Abbreviations

SPECT: single-photon-emission computed tomography.

## Competing interests

The authors declare that they have no competing interests.

## Consent

Written informed consent was obtained from the patient for publication of this case report and any accompanying images.

## Authors' contributions

AAG, AH and YAO were the physicians responsible for the care of the patient. SH and FA were involved in executing and analyzing neuro-imaging data. SA reviewed the relevant literature and provided the neuropsychological underpinning of the case. All the authors contributed to writing of the paper and the editing of the final manuscript before submission.
